# Assessing a Potential Role of Host Pannexin 1 during *Chlamydia trachomatis* Infection

**DOI:** 10.1371/journal.pone.0063732

**Published:** 2013-05-20

**Authors:** Mary J. McKuen, Gerhard Dahl, Kenneth A. Fields

**Affiliations:** 1 Departments of Microbiology and Immunology, University of Miami Miller School of Medicine, Miami, Florida, United States of America; 2 Departments of Physiology and Biophysics, University of Miami Miller School of Medicine, Miami, Florida, United States of America; University of California Merced, United States of America

## Abstract

Pannexin 1 (Panx1) is a plasma membrane channel glycoprotein that plays a role in innate immune response through association with the inflammasome complex. Probenecid, a classic pharmacological agent for gout, has also been used historically in combination therapy with antibiotics to prevent cellular drug efflux and has been reported to inhibit Panx1. As the inflammasome has been implicated in the progression of *Chlamydia* infections, and with chlamydial infections at record levels in the US, we therefore investigated whether probenecid would have a direct effect on *Chlamydia trachomatis* development through inhibition of Panx1. We found chlamydial development to be inhibited in a dose-dependent, yet reversible manner in the presence of probenecid. Drug treatment induced an aberrant chlamydial morphology consistent with persistent bodies. Although Panx1 was shown to localize to the chlamydial inclusion, no difference was seen in chlamydial development during infection of cells derived from wild-type and Panx1 knockout mice. Therefore, probenecid may inhibit *C. trachomatis* growth by an as yet unresolved mechanism.

## Introduction

*Chlamydiae* are obligate intracellular pathogens that preferentially replicate within mucosal columnar epithelial cells. All chlamydial species exhibit a unique biphasic developmental cycle. The cycle is initiated when host cells are invaded by the extracellular, metabolically inactive elementary body (EB). Internalized EBs are enveloped in a parasitophosphorus vacuole termed an inclusion. The inclusion represents a specialized intracellular niche that enables chlamydial survival by segregating the bacteria from host defense mechanisms while enabling trafficking pathways that provide essential nutrients [1]. Within the inclusion, EBs undergo primary differentiation into metabolically active, yet non-infectious reticulate bodies (RBs). Following replication, RBs undergo asynchronous secondary differentiation back into EBs and exit the cell to begin a second round of infection in neighboring cells [2]. Development can be arrested *in vitro* by treatments such as IFNγ, β-lactam antibiotics, or iron deprivation [3]. In each case, chlamydiae enter a “persistent” state in which abnormally enlarged RBs fail to differentiate back into EBs [4].

Sexually-transmitted genital infections of *Chlamydia trachomatis* represent the most common infectious disease reported to the Centers for Disease Control. The 1.3 million cases reported in 2010 are considered an underrepresentation due to non-detection and non-reporting [5]. Although the sexually transmitted infection is usually asymptomatic, sequelae such as urethritis in men and women, and cervicitis in women can manifest [6]. Untreated genital chlamydial infection ascends the upper reproductive tract, which can progress to epidiymitis and proctitis in men, and pelvic inflammatory disease (PID) and salpingitis in women [7].

In cases where chlamydial infections are successfully diagnosed, antibiotic treatment with doxycycline or, more recently, azithromycin typically results in resolution of infections. Historically, probenecid was used in combination therapy with antibiotics to augment their potency by blocking cellular drug efflux and thereby enhancing intracellular pharmacological concentrations [8], [9]. Indeed, ampicillin augmented with probenecid represented a common approach for treatment of polymicrobial PID cases [10]. Beta-lactam antibiotics have limited efficacy in successful treatment of chlamydial genital infections [11] and the combinatorial therapy was similarly found to be ineffective in resolving chlamydial infections [12]. A mainstay treatment for gout, probenecid has been shown to function by inhibition of membrane transporters including those for organic anions (OAT) [13], [14], drug efflux [15] and more recently, pannexin 1 (Panx1) [16].

Panx1 is a transmembrane glycoprotein that forms channels containing 6 subunits [17]. Nearly ubiquitous in all tissue types, Panx1 is involved in a variety of cellular responses, including the innate immune response, apoptosis, cellular differentiation, tumorigenesis, and paracrine signaling [18], [19]. The Panx1 channel is relatively non-selective, allowing passage of anions, cations, dyes, and ATP [19]–[22]. Although discovered as a gap junction protein, the stimulation (via purinergic receptors) and subsequent inhibition of Panx1 channels by ATP has led to the characterization of the channel as an ATP release channel [19], [22]–[24]. Panx1 manifests effects on apoptosis, pyroptosis, and innate immune response via intimate association with the P2X_7_ receptor [25]–[29]. Furthermore, Panx1 has been suggested to be part of the inflammasome complex through co-precipitations with P2X_7_ receptor, as well as with inflammasome components such as NLRP1 (NACHT, LRR, and PRY domains-containing proteins), ASC (apoptosis-associated speck-like protein containing a CARD), caspases 1 and 11, and XIAP (X-linked inhibitor of apoptosis protein) [30].

The inflammasome has been shown to be important in the innate immune response to chlamydial infection via NLRP3 and ASC dependent activation of caspase 1 [31]–[33]. Pharmacological studies targeting NLRP3 resulted in a dose-dependent inhibition of *C. pneumoniae* infection in monocytes [34]. While IL-1β secretion has been implicated as important in clearance, it has been shown to be minimally important in *C. muridarum* infection while ASC and NLRP3 may have an IL-1β independent importance in clearance [32], [33], [35]. Since the host inflammasome is relevant to both *Chlamydia* and Panx1 biology, we chose to investigate whether probenecid would directly affect chlamydial development through inhibition of Panx1. We found that probenecid treatment directly inhibited chlamydial development in a dose-dependent and reversible manner, yet the mechanism of probenecid inhibition appears to be independent of Panx1.

## Results and Discussion

### Inhibition of *C. trachomatis* Development by Probenecid

**Figure 1:**
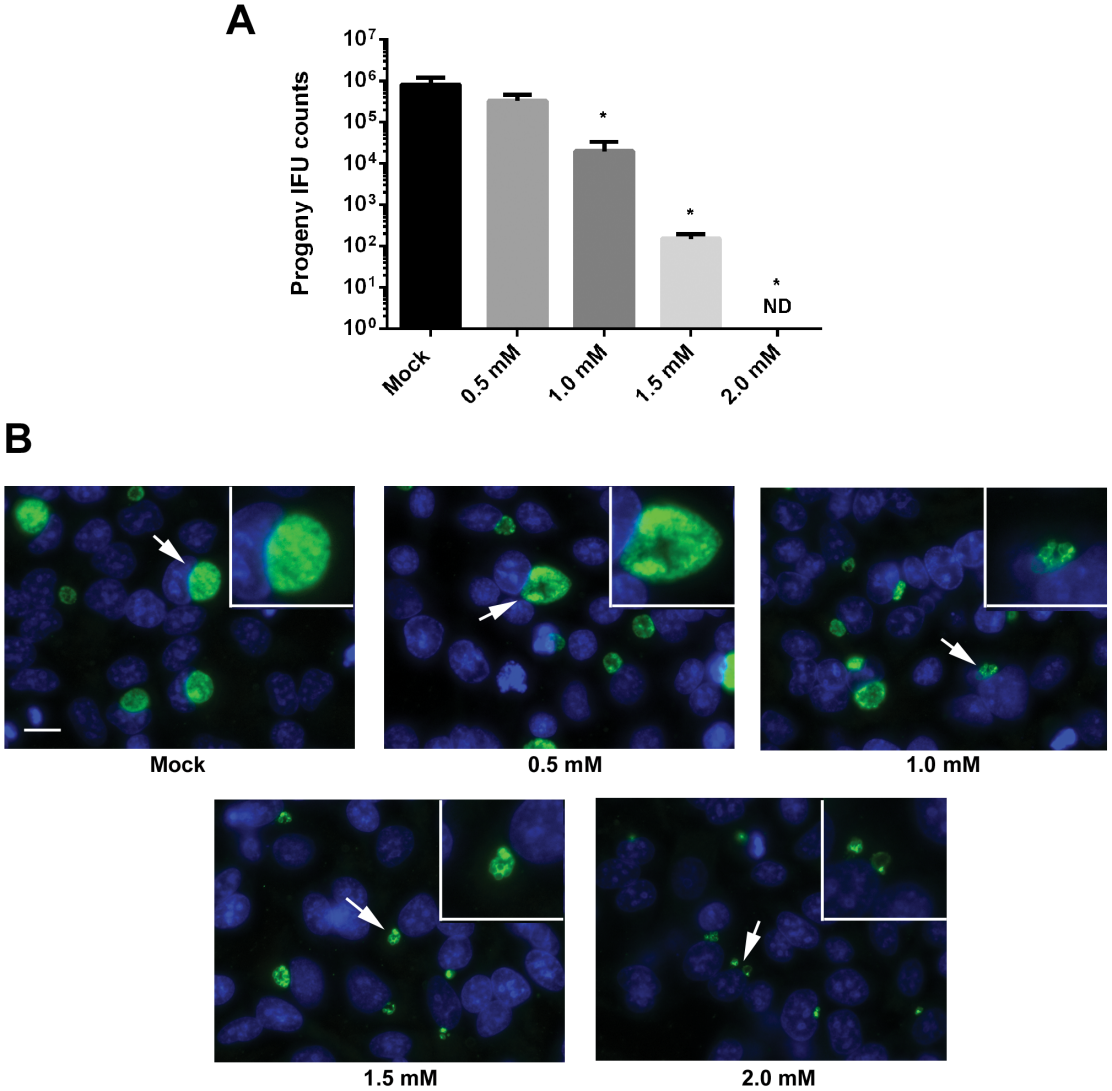
Dose-dependent inhibition of *C. trachomatis* growth by probenecid. HeLa cells were infected at an MOI 1. Cells were mock treated or treated with probenecid at 0.5, 1.0, 1.5, or 2.0 mM final immediately after infection. Cultures were maintained with the respective treatment for 24 hr and disrupted for second passage progeny counts (A) or fixed and stained for immunofluorescence visualization of inclusions (B). Data for progeny counts are represented as mean ± standard deviation of duplicate samples, and One-Way ANOVA analysis was used to compare probenecid treatments with the mock sample (P<0.05). No progeny were detected (ND) from cultures containing 2.0 mM probenecid. For indirect immunofluorescence, chlamydiae were detected by probing with α-HSP60 followed by Alexa 488-conjugated secondary antibodies (green) and host cells were visualized by DAPI staining of nuclei (blue). Epi-fluorescence images were acquired at 90× magnification and relative magnification of insets was maintained for each treatment. Arrows indicate area of inset and Bar = 5 µm.

*C. trachomatis* growth can be quantitatively examined via enumeration of accumulating infectious forming units (IFUs). We therefore tested the ability of probenecid to interfere with chlamydial development by measuring the levels of progeny chlamydiae in the presence of increasing concentrations of probenecid (Fig. 1A). HeLa monolayers were infected with *C. trachomatis* serovar L2 in the absence of drug. Immediately after infection, cultures were supplemented with medium alone (Mock) or containing probenecid to achieve final concentrations ranging from 0.5 mM to 2.0 mM. Cultures were maintained for 24 hr prior to disruption for progeny counts. We observed a dose-dependent decrease in recoverable IFUs after treatment with probenecid with no progeny being detected at the highest (2.0 mM) concentration. Identical IFU patterns were detected when HeLa cells were additionally pretreated with probenecid prior to infection with *C. trachomatis* (data not shown). Hence the observed decrease in *Chlamydia* IFUs is most likely due to an effect on the bacteria after invasion of host cells. Probenecid is an established inhibitor of Panx1 activity in a variety of cell types and uptake of the dye YoPro-1 has been used to verify pharmacologic inhibition of Panx1 [16]. We therefore verified that probenecid inhibited panx1 in HeLa cells by examining dye exclusion in the presence or absence of inhibitor (Fig. S2). As expected, treatment of cultures with probenecid reduced cell uptake of YoPr-1. Hence, our treatment with probenecid was sufficient to impact pannexin activity in these cells.

Parallel cultures were examined by indirect immunofluorescence to directly visualize the nature of probenecid-mediated inhibition (Fig. 1B). Representative epi-fluorescence microscopy fields of view are presented where chlamydial inclusions are shown in relation to DAPI-stained host nuclei. In agreement with IFU data, a dose-dependent decrease in inclusion size was apparent when probenecid was present at concentrations greater than 1.0 mM. Chlamydial trafficking was not perturbed since all inclusions were localized to the peri-nuclear region of infected cells. Infections carried out at 4°C to synchronize invasion did not alter these results (data not shown). Overt alterations in inclusion morphology were detected at probenecid concentrations of 1.0–2.0 mM where digitally zoomed images revealed atypically large RBs. Although enlarged bodies were detected at all of the higher probenecid concentrations, replication became evidently decreased since inclusions in 2.0 mM probenecid cultures appeared to contain only a few bacteria. These data therefore indicate that treatment with probenecid can effectively and directly interfere with *C. trachomatis* development.

**Figure 2:**
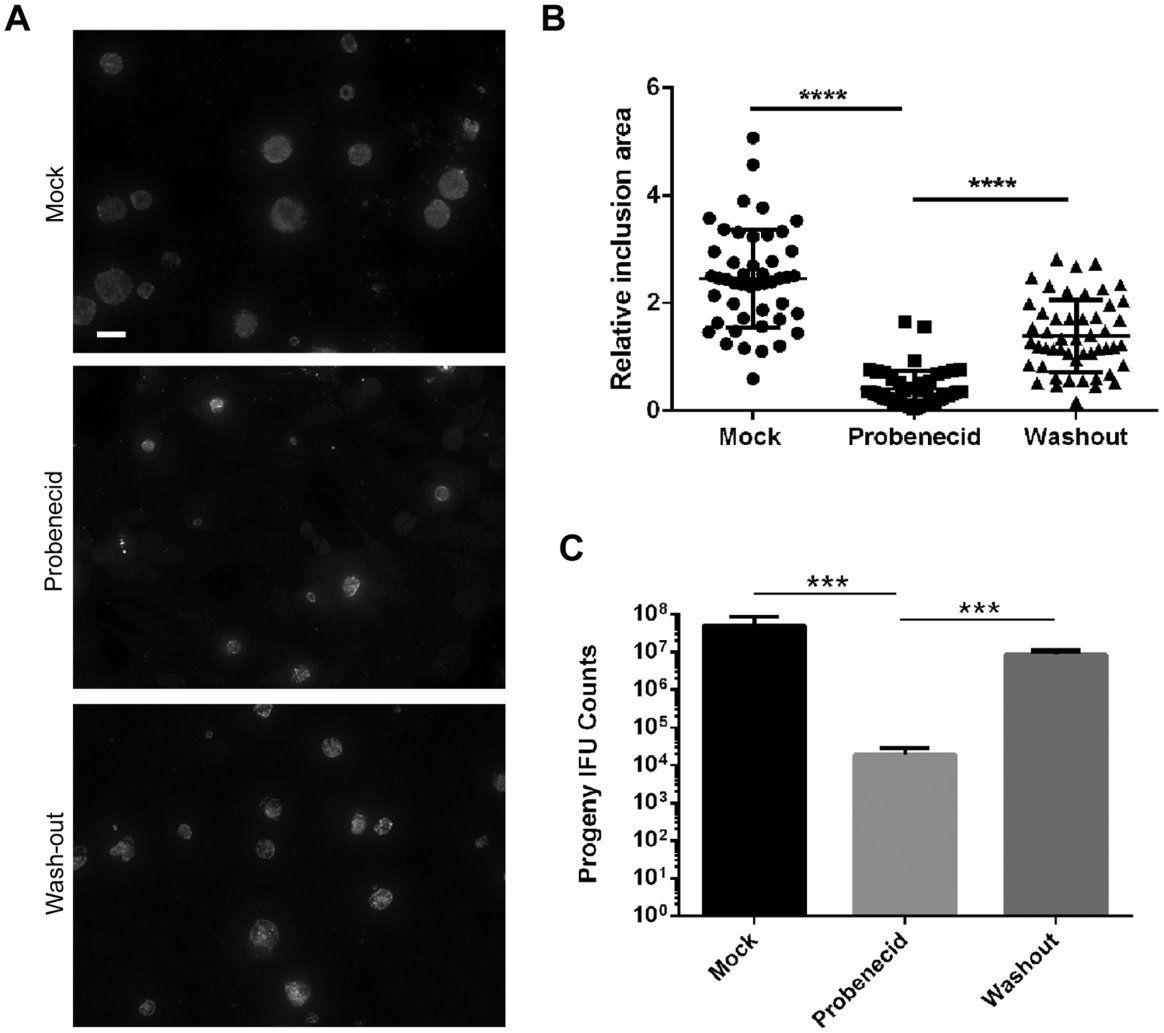
Probenecid-mediated growth inhibition is reversible. HeLa cells were infected with *C. trachomatis* at an MOI = 1 and mock treated or treated with 2.0 mM probenecid immediately after infection. At 24 hr post infection, probenecid was washed away from one replicate and replaced with medium lacking drug (washout). All cultures were incubated an additional 24 hr and parallel cultures were fixed for immunofluorescence or disrupted for progeny counts. (A) Inclusions were stained with α-HSP60 and representative fields of view from each culture are shown. Bar = 5 µM. (B) Representative inclusion areas were computed and represented individually. A student’s T-test was performed to indicate statistical significance of differences (****; P<0.0001). (C) Data for progeny counts are represented as mean ± standard deviation of triplicate samples, and a student’s T-test was used to address significance (***; P<0.001).

We extended our analysis to 48 hr post infection to gauge whether chlamydial growth was solidly blocked or simply slowed. Direct examination of chlamydial inclusions cultivated in the presence of 2.0 mM probenecid displayed a marked reduction in apparent size (Fig. 2A) and inclusion area (Fig. 2B) compared to the mock-treated control. However, it was clear that inclusions contained multiple bacteria, raising the possibility that completion of the developmental cycle can occur, at albeit lower levels. This was directly tested via quantitation of progeny EBs in parallel cultures (Fig. 2C). As expected, a statistically significant (P<0.001) reduction of progeny counts was detected in cultures maintained in 2.0 mM probenecid. This reduction was reversible since washout of probenecid at 24 hr resulted in a significant (P<0.001) increase in progeny EBs compared to non-washout cultures. Collectively, these data indicate that probenecid treatment is not chlamydiacidal. Instead, the developmental cycle is perturbed leading to decreased levels of replication.

### Probenecid Treatment Induces Persistent Chlamydiae

**Figure 3:**
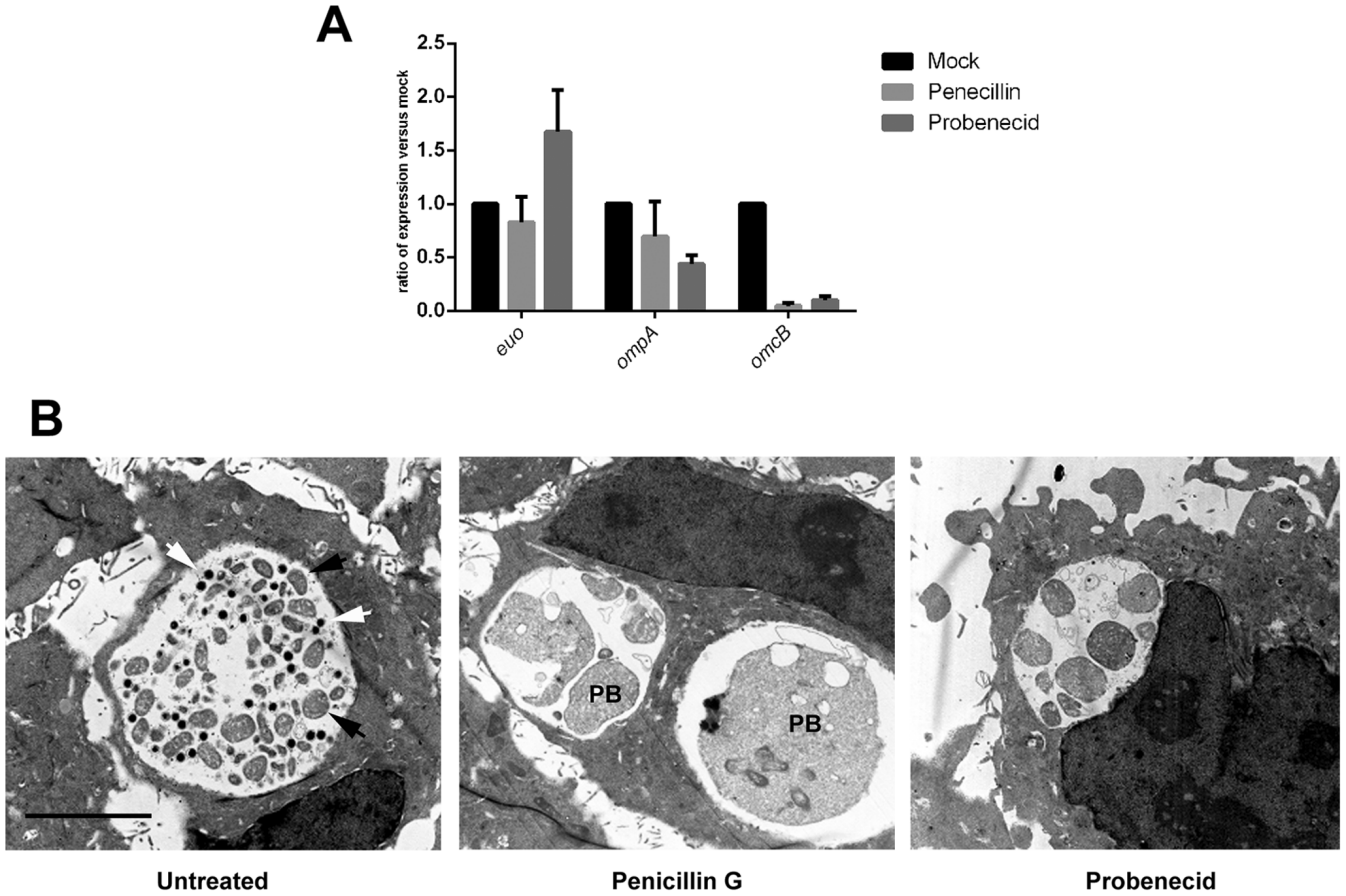
Evidence of persistent growth in the presence of probenecid. HeLa cells were infected with *C. trachomatis* at an MOI = 1 and either mock treated or treated with 100 U/ml of penicillin G or 2.0 mM probenecid immediately after infection. (A). Cultures were processed for transmission EM analysis at 24 hr post infection. All images were acquired at a magnification of 4600 and representative micrographs are shown. Open arrows indicate typical EBs, closed arrows RBS, and representative persistent bodies are indicated (PB). Bar = 5 µM. (B). Whole culture RNA was harvested at 24 hr post infection and message levels for euo, ompA, and omcB were assessd via qRT-PCR. Values are reported for each gene as a ratio of mock versus inhibitor treatment.

The detection of abnormally large chlamydiae and reversibility of inhibitory effects suggested that probenecid may induce persistent chlamydial growth. Although some molecular indicators of persistent growth have been suggested, detection of consistently enlarged bodies via electron microscopy is considered the most reliable evidence in determining persistence [3]. We therefore compared bacteria in probenecid- and mock-treated cultures that were processed for electron microscopy at 24 hr post infection (Fig. 3A). Treatment of chlamydial cultures with penicillin G induces a robust persistent state [3] and was used as a positive control for persistent bodies. As anticipated, mock-treated cultures contained inclusions with both small, electron-dense EBs and slightly larger RBs whereas only very large persistent bodies were detected in penicillin-treated cultures. Probenecid treatment yielded an intermediate phenotype. Enlarged RB bodies were predominantly detected whereas EBs were not readily apparent. These data are consistent with the previous (Fig. 1A) lack of detection of progeny chlamydiae at 24 hr and are consistent with probenecid inducing persistent growth of *C. trachomatis*. Gene expression analyses during persistent growth have indicated a lack of expression for late-cycle genes [3]. We therefore examined message levels for early-cycle (euo), mid-cycle (opmA) and late-cyle (omcB) genes [36]. RT-PCR analyses (Fig. 3B) revealed that gene expression in probenecid-treated cultures closely mirrored those seen in the presence of penicillin. Importantly, levels of omcB were reduced compared to mock-treated control and were consistent with a lack of accumulating EBs during persistent growth. We were not able to restore chlamydial growth in the presence of probenecid by addition of exogenous iron or tryptophan (data not shown) suggesting an alternative mechanism leading to a persistent state.

### Multiple Pannexin-inhibiting Drugs also Affect Chlamydial Growth

**Figure 4:**
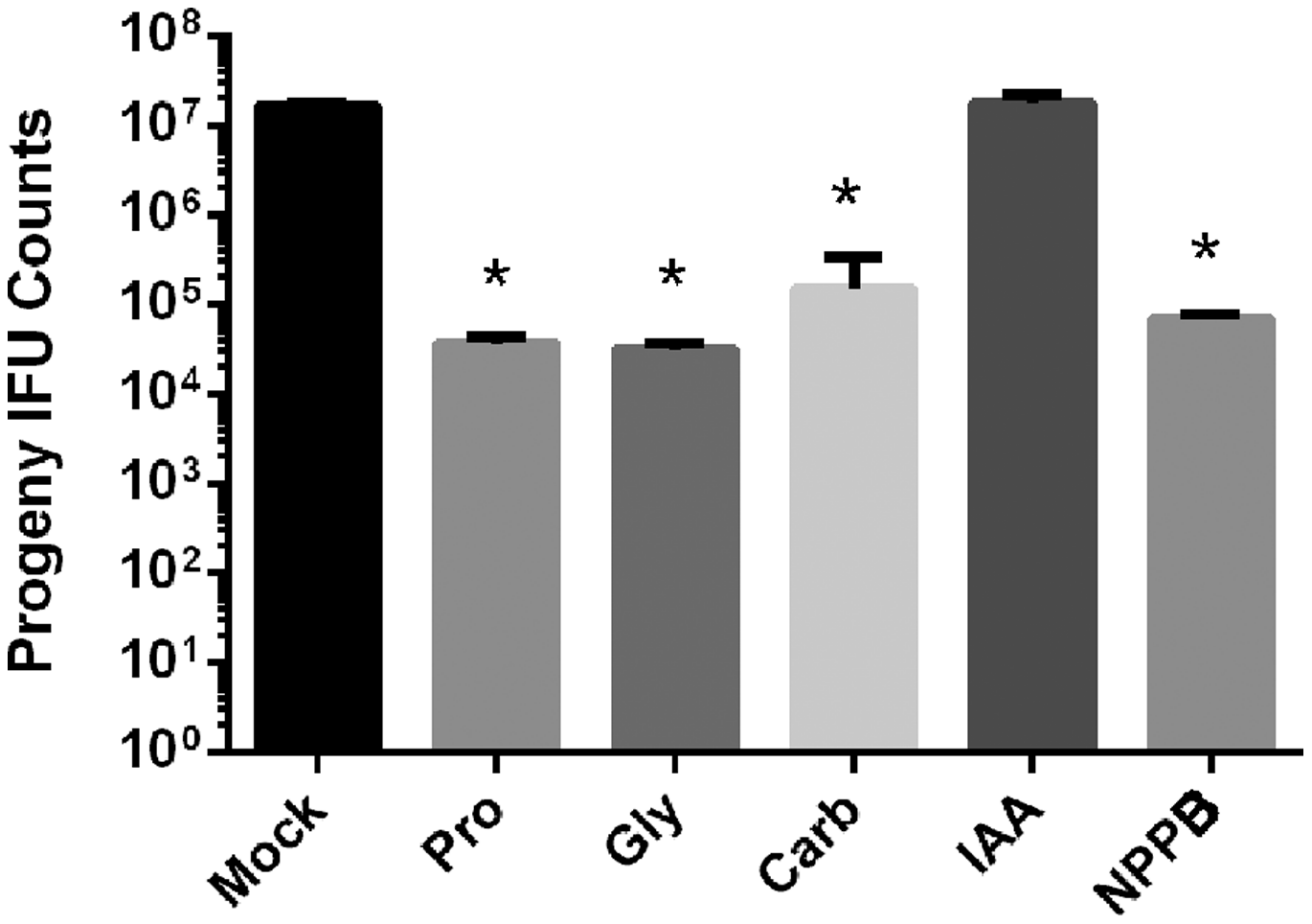
Inhibition of chlamydial development with a panel of pannexin-inhibitory drugs. *C. trachomatis* infected HeLa cells were maintained with 1.5 mM probenecid (Pro), 75 µM glyburide (Gly), 75 µM carbenoxolone (Carb), 100 µM IAA, or 100 µM NPPB for 24 hr. Data for progeny counts are represented as mean ± standard deviation of duplicate samples, and One-Way ANOVA analysis was used to compare drug treatments to the mock control (*; P<0.05).

Established direct targets of Probenecid include channel proteins such as organic anion transporters [13], [14], MDR transporters [15], and the channel Panx1 [16]. Given that the probenecid concentrations required to inhibit chlamydial growth correlated with those affecting Panx1, we reasoned that inhibition of Panx1 was the most likely to manifest as decreased chlamydial development. This hypothesis was initially tested by assessing the susceptibility of chlamydiae to additional drugs known to inhibit Panx1 (Fig. 4). *C. trachomatis*-infected HeLa cultures were treated with glyburide (Gly), carbenoxolone (Carb), indanyloxyacetic acid 94 (IAA), or 5-nitro-2(3-phenylpropylamino)-benzoic acid (NPPB) at concentrations established to inhibit Panx1 [16], [37], [38]. These treatments were compared to mock or probenecid-treated cultures as relevant controls. Progeny counts from Gly-, Carb-, and NPPB-treated cultures were comparable to probenecid-treatment and were significantly (P<0.05) decreased compared to the mock control. IAA treatment did not affect chlamydial growth since progeny counts were not different from the mock control. In addition, inclusions in IAA-treated cultures were indistinguishable from mock-treated controls (data not shown). With the exception of IAA, inhibition of chlamydial growth with the remaining drugs was consistent with Panx1 activity being required for optimal chlamydial development.

### Recruitment of Pannexin I to the Chlamydial Inclusion

**Figure 5:**
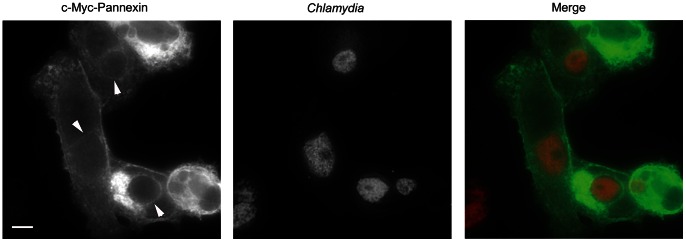
Pannexin 1 co-localization with chlamydial inclusions. HeLa cells were transfected with c-myc-Pannexin 1-expressing plasmid and subsequently infected with *C. trachomatis*. Cultures were fixed at 24 hr post infection and stained for chlamydiae (red) or Panx1 (green). Arrows indicate inclusion membrane-specific signal and Bar = 5 µM.

Panx1 is a transmembrane, pore-forming protein and could have a role in chlamydial infection at either the plasma or inclusion membranes. Panx1 localization was examined to further explore a potential role of Panx1 in chlamydial survival (Fig. 5). Unfortunately, we were unable to detect endogenous Panx1 via indirect immunofluorescence with commercially available antibodies. Although Panx1-specific antibodies raised in chickens [22] did reveal significant signal at the inclusion membrane, the data were difficult to interpret since the pre-immune serum cross-reacted strongly with chlamydiae (Fig. S3). Hence, localization was visualized with myc-specific antibodies in cells exogenously expressing myc-tagged Panx1. Transfected HeLa cells were infected with *C. trachomatis* and processed for indirect immunofluorescence at 24 hr post infection. We routinely detected Panx1 in both the plasma membrane and in rim-like patterns encircling chlamydiae. This pattern is indicative of inclusion membrane localization typically observed with chlamydial Inc proteins [39] and indicates that Panx1 is recruited to the *Chlamydia*-containing parasitophorous vacuole. Hence, Panx1 could contribute to maintaining an intracellular niche at either the plasma or inclusion membranes, or at both membranes.

### Chlamydial Growth in Panx1−/− Host Cells

**Figure 6:**
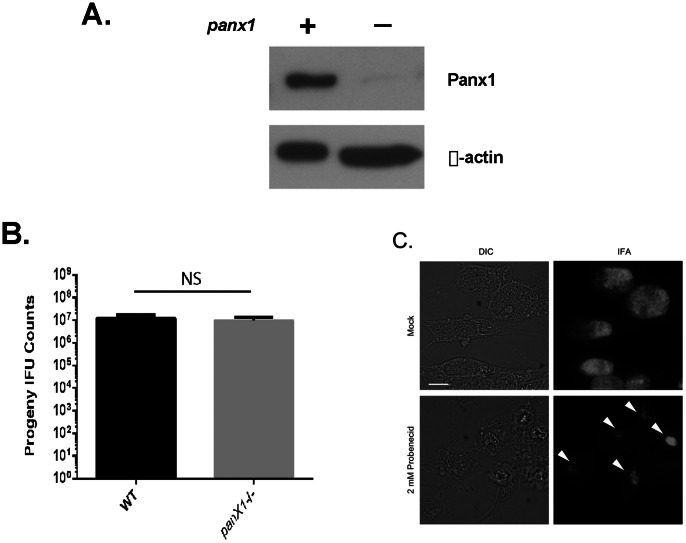
Growth of *C. trachomatis* in Panx1-deficient cells. Panx1 levels in *panx1−/−* astrocytes were examined via immunoblot in comparison with panx1-expressing cells. Levels of β-actin were assessed as loading controls. (B) Astrocytes prepared from wild-type and *panx1*−/− mice were infected with *C. trachomatis* for 24 hr. Data for progeny counts are represented as mean ± standard deviation of duplicate samples. (C) *panx1*−/− astrocytes were infected with *C. trachomatis* and mock treated or treated with 2 mM probenecid. Inclusions were visualized by DIC or immunofluorescence staining 24 hr post infection. Bar = 5 µM.

Although multiple Panx1-inhibiting drugs interfered with chlamydial growth, pharmacologic studies are not definitive indicators of function. Many drugs have multiple targets that cannot be excluded as contributing to observed phenotypes. For example, NPPB also inhibits chloride ion channels [40]. Moreover, glyburide-mediated inhibition of chlamydial growth was recently attributed to inhibition of lipid transport to the chlamydial inclusion [41]. Therefore, we wanted to definitively test the requirement of Panx1 in productive chlamydial growth by infecting cells deficient in Panx1. We used astrocytes from these mice since that cell type readily supported chlamydial growth and was reported to not express Panx1 [24]. Our immunoblot analysis of these cells indicated a lack of wild-type Panx1 levels (Fig 6A). However, a low-abundance band was routinely detectible in the panx1−/− astrocytes that migrated near WT Panx1. Interestingly, no difference was detected in progeny counts from *C. trachomatis* infected cultures of WT and knock-out cells (Fig. 6B). We further tested for a Panx1 requirement by assaying the susceptibility of chlamydiae to probenecid in these cells (Fig. 6C). Unexpectedly, chlamydial inclusions were markedly smaller and contained enlarged chlamydiae in the probenecid treatment, indicating that chlamydiae remained susceptible to probenecid in *Panx1*−/− cells. These data therefore indicate that the growth deficiency mediated by probenecid most likely occurs independently of Panx1.

Probenecid has multiple molecular targets, raising the possibility that the observed detrimental impact on chlamydial development could be manifested via inhibition of anion transporters. However, it is unclear whether these proteins are expressed in HeLa cells. Moreover, pharmacological studies of probenecid action indicate that concentrations significantly lower than 0.5 mM are sufficient to mediate inhibition of OAT [42] and MDR [15] transporters. Since 0.5 mM did not have a significant impact on chlamydial growth, we regard these other potential targets as unlikely. Based on our immunoblot analysis of astrocytes, it is formally possible that low levels of Panx1 exist in these astrocytes. Importantly, however, electrophysiology studies have shown that these cells lack detectable Panx1function [24], making it unlikely that Probenecid-mediated affects are manifested via inhibition of Panx1 in these cells. We also cannot exclude the existence of a probenecid-sensitive protein with redundant function. We feel that the most likely explanation is that probenecid-mediated inhibition of chlamydial growth is manifested through a currently unappreciated molecular target. The most direct evidence to this conclusion is that the Panx1-inhibiting drug IAA did not affect chlamydial growth. However, we acknowledge the possibility that IAA may not be able to gain access to inclusion-localized Panx1. Finally, it is possible that probenecid could interfere with chlamydial growth by directly targeting a chlamydial protein. This is true for all pharmacologic agents that target host molecules and inhibit chlamydial growth. For example, the mammalian metalloprotease inhibitor GM6001 also inhibits chlamydial growth [43]. However, the chlamydial growth inhibition is manifested by targeting the chlamydial peptide deformylase [44].

Regardless of the role of probenecid, the recruitment of Panx1 to the chlamydial inclusion is interesting and implies a function at the interface between microbe and host. One possibility is that an open Panx1 channel could allow anions and other small metabolites to gain access to the lumen of the inclusion. Studies have indicated that the inclusion membrane has limited permeability [45] but does allow passage of ions including K+ [46]. This is an intriguing possibility since panx1 allows transport of K+ across host membranes [19]. Finally, we have shown that supplementation of antibiotic therapies with probenecid could have the undesired effect of inducing chlamydial forms that could persist or perpetuate an infection.

## Methods

### Cell Culture and Organisms

These studies employed HeLa 229 (CCL 21; American Type Culture Collection, Manassas, VA) epithelial cells or astrocytes derived from *Panx1*−/− mice as described [24]. As previously noted (24), all mice were maintained at Albert Einstein College of Medicine in accordance with IACUC-approved protocols. Specific animal protocols were approved by the Albert Einstein Animal Care and Use Committee. Eukaryotic cell cultures were routinely maintained at 37°C in the presence of 5% CO_2_/95% humidified air in RPMI-1640 medium (Invitrogen, Carlsbad, CA) supplemented with 10% (vol/vol) fetal bovine serum (Sigma-Aldrich, St. Louis, MO) and 10 µg/ml gentamicin (Invitrogen). Cells were infected with density gradient-purified *C. trachomatis* LGV-434, serotype L2 in Hank’s balanced salt solution (HBSS; Invitrogen) at 37°C as previously described [47], [48]. Infected cells were then incubated for respective times at 37°C in the presence of 5% CO_2_/95% humidified air. Where indicated, cultures were supplemented after infection with penicillin G (Sigma) at 100 U/ml or [4-(dipropylsulfamoyl)benzoic acid] (probenecid; Sigma) glyburide (Sigma), carbenoxolone (Sigma), indanyloxyacetic acid 94 (IAA; Sigma), or 5-nitro-2(3-phenylpropylamino)-benzoic acid (NPPB; Tocris Bioscience, Bristol, UK) at indicated concentrations. Where appropriate, primary cultures were lysed at indicated times in cold water, diluted into HBSS, and re-plated onto fresh HeLa cells for progeny inclusion forming units (IFUs) as described [49].

### YoPro-1 Dye Exclusion

HeLa cells were grown to confluency in a 96 well plate (Corning) in 200 µl of RPMI (Invitrogen-Gibco). Media was replaced with 100 µl OR2 buffer (Ca^2+^ -free oocyte solution: in mM: 82.5 NaCl, 2.5 KCl, 1.0 MgCl_2_, 1.0 CaCl_2_, 1.0 Na_2_HPO_4_, and 5.0 HEPES, pH 7.5) for mock treatment or OR2 containing 1 mM probenecid as described [16]. Cells were treated at room temperature for 10 minutes. 50 µl of solution was removed from the wells and replaced with 0.5 µM YoPro-1 iodide (Invitrogen) in 50 µl OR2, water, or water with 1.0 mM probenecid. Images were acquired 1 minute after stimulation at 40x magnification with a Canon DP12-2 digital microscope camera on an Olympus CKX41 inverted fluorescence microscope.

### RT-PCR Methods

HeLa cells were grown to confluency in triplicate per treatment in a 6 well plates (Corning) in RPMI (Invitrogen-Gibco) containing 10% heat-inactivated fetal bovine serum (Sigma). Cells were mock infected or infected with DG purified *Chlamydia trachomatis* serovar L2 at a MOI of 0.1 in 1 mL SPG at 37°C for 1 hour. Inoculum was replaced with 2 mL RPMI (mock) or treated with RPMI containing 2 mM probenecid or 100 units/mL penicillin G for 24 hours at 37°C with 5% CO_2_. RNA was harvested with Aurum Total RNA Mini Kit (Biorad). Gene expression of *ompA, euo,* and *omcB* (normalized based on *rpoD* levels) was analyzed by RT-PCR using custom primers (Fig. S1). Reactions were performed with an iScript One-Step RT-PCR kit with SYBR Green (Biorad) on a CFX96 Real-Time PCR Detection System (Biorad).

### Transmission Electron Microscopy (TEM)

HeLa cells were grown to semi-confluence on 13 mm Thermonox (Nunc, Naperville, IL) coverslips and infected with *C. trachomatis* as described [50]. Infected monolayers were incubated at 37°C in RPMI or RPMI supplemented with either probenecid or penicillin G. Cultures were then fixed at 24 hr post infection with 4% (wt/vol) paraformaldehyde/2.5% (vol/vol) glutaraldehyde in 100 mM sodium cacodylate, pH 7.4. Cells were post fixed in 2% osmium tetroxide in 0.1 M phosphate buffer, dehydrated through a series of graded ethanols, and embedded in EM-bed (Electron Microscopy Sciences, Fort Washington, PA). 80 nm sections were cut on a Leica Ultracut-R ultramicrotome and stained with uranyl acetate and lead citrate. The grids were viewed at 80 kV on a Philips CM-10 transmission electron microscope and images captured by a Gatan ES1000W digital camera.

### Immunodetection

The presence of pannexin 1 was examined by immunoblot of eukaryotic cells. Whole-culture extracts were generated by lysis of confluent cell monolayers in ice-cold water containing Complete^tm^ protease inhibitor cocktail (Roche Diagnostic, Indianapolis, IN). Proteins were concentrated by the addition of trichloroacetate (TCA; Fischer Scientific, Suwanne, GA) to 10% (vol/vol), and precipitate proteins were solubilized in electrophoresis sample buffer [2.3% (wt/vol) sodium dodecyl sulfate (SDS), 5% (vol/vol) β-mercaptoethanol, 25% (vol/vol) glycerol, and 60 mM Tris pH 6.8]. Material was resolved in polyacrylamide gels [12% (vol/vol) polyacrylamide] by SDS-PAGE and transferred to Immobilon-P (Millipore Corp., Deford, MA) for probing with anti-β actin (Sigma) or anti-Pannexin1 (Invitrogen). Proteins were detected by probing with horseradish peroxidase-conjugated IgG (Sigma) followed by development with ECL Plus chemiluminescent substrate (GE Healthcare, Buckinghamshire, UK).

*C. trachomatis* inclusions were visualized in infected HeLa monolayers by indirect immunofluorescence. HeLa monolayers were cultivated on 12 mm coverslips and infected at a calculated MOI no greater than 1. Cultures were fixed and permeablized with methanol, blocked with phosphate buffered saline (PBS; 135 mM NaCl, 2.7 mM KCl, 10 mM Na_2_HPO_4_, 1.8 mM KH_2_PO_4_; pH 8.0) supplemented with 5% (wt/vol) BSA and 0.05% (vol/vol) Tween-20 (Sigma-Aldrich), and probed with α-HSP60 (Santa Cruz Biotechnology, Santa Cruz, CA) followed by fluorophore-conjugated α-mouse IgG antibodies (Molecular Probes, Inc., Eugene, OR). For studies investigating the localization of pannexin 1, HeLa monolayers were first transfected with pRK5 expressing c-myc-Pannexin 1 [17] with lipofectamine 2000 according to the manufacturer’s instructions (Invitrogen). Cultures were infected with chlamydiae 6 hr later and cultures were processed for microscopy at 24 hr post infection. Epitope-tagged Pannexin 1 was detected using c-myc-specific antibodies. Epi-fluorescence images were acquired on a TE2000U inverted photomicroscope (Nikon® Inc., Melville, NY) equipped with a Retiga EXi 1394, 12 bit monochrome CCD camera (QImaging, Surrey, BC, Canada) and MetaMorph imaging software version 6.3r2 (Molecular Devices, Downingtown, PA). Where appropriate, inclusion areas were computed from acquired images using the MetaMorph Region Measurements function. 50 inclusions were measured from representative images that were appropriately scaled. All images directly used in figures were equivalently processed using Adobe Photoshop® CS2 version 9.0 (Adobe Systems, San Jose, CA).

### Statistical Analyses

Presented data are representative of a minimum of three experimental replicates. Statistical analyses were performed using GraphPad Prism 6 (Graphpad Software, San Diego, CA).

## Supporting Information

Figure S1 **RT-PCR primers used in this study.** Primer pairs are listed as either sense (FWD) nonsense (REV).
(TIF)

Figure S2 **Probenecid-mediated inhibition of Panx1 activity in HeLa cells.** Cells were mock treated (unstimulated) or water stimulated in the presence (stimulated+probenecid) or absence (stimulated) of 1.0 mM probenecid. Live-cell phase contrast and corresponding fluorescence images were taken 1 min after addition of YoPro-1 iodide.
(TIF)

Figure S3 **Immunolocalization of endogenous Panx1 in** ***C. trachomatis*** **infected cells.** HeLa cells were infected at an MOI of 1 and fixed 24 hr post infection. Parallel samples were probed with chicken anti-pannexi1 (Immune) or matched pre-immune (Pre-immune) serum. Panx1 was visualized via epi-fluorescence microscopy after probing with Alexa594-coupled secondary antibodies. Images were acquired at 90X magnification and Bar = 5 µm. Chlamydial inclusions (I), host nuclei (N), or Panx1 colocalization with inclusions (arrows) are indicated. Pre-immune serum alone resulted in detection of intracellular chlamydiae.
(TIF)
