# Supplementary figures and images for: Assessing a Potential Role of Host Pannexin 1 during Chlamydia trachomatis Infection
Source: PLoS One. 2013 May 20;8(5):e63732. doi: 10.1371/journal.pone.0063732 (PMC3659042; doi:10.1371/journal.pone.0063732)

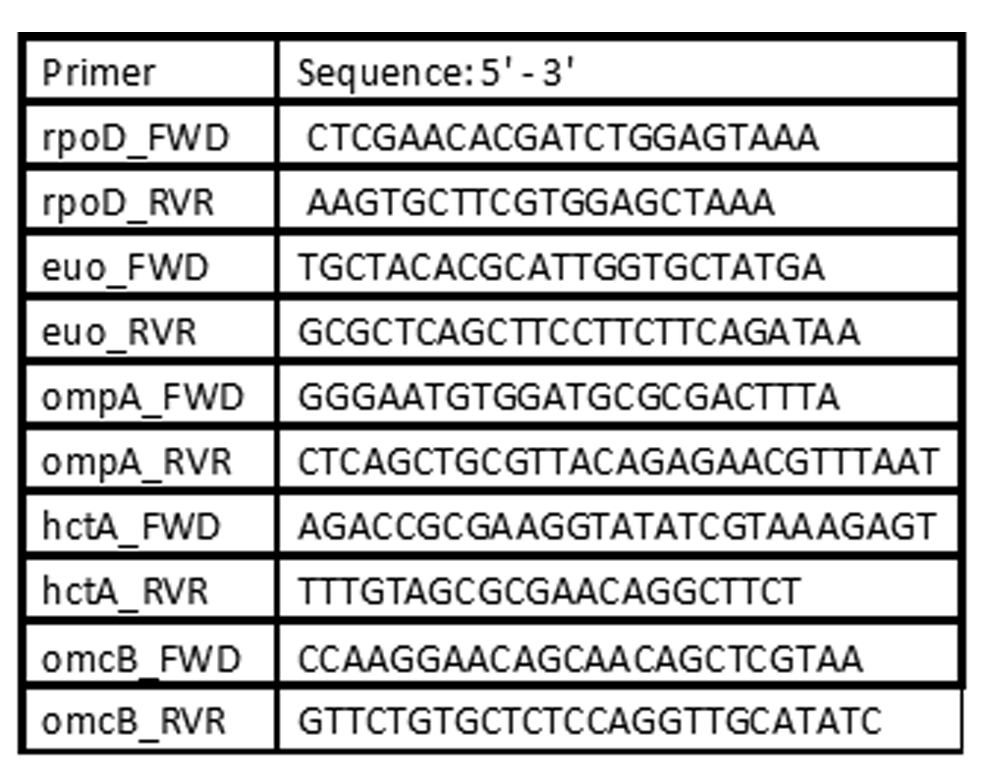

Supplement: Figure S1 — RT-PCR primers used in this study. Primer pairs are listed as either sense (FWD) nonsense (REV). (TIF) [file pone.0063732.s001.tif]

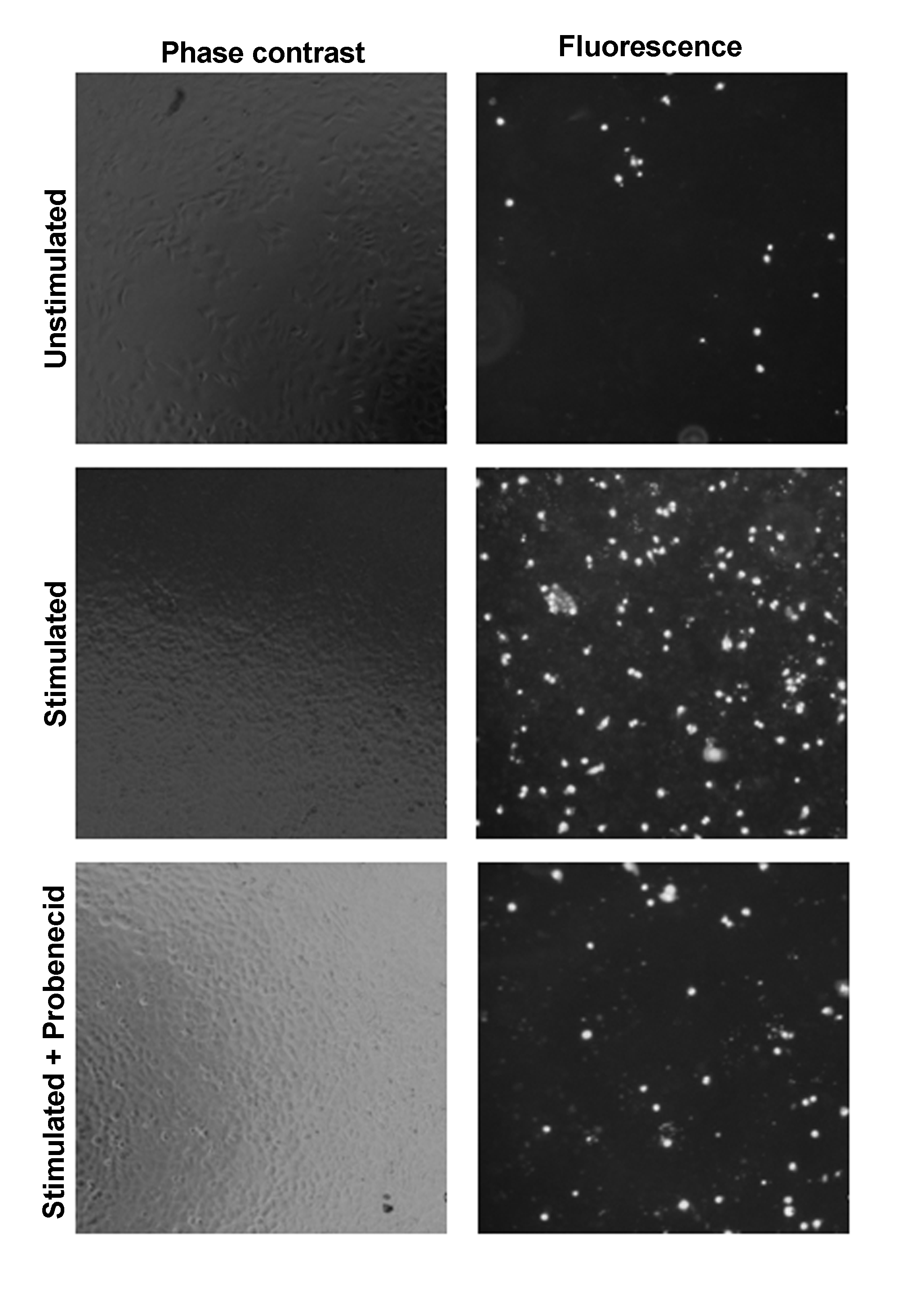

Supplement: Figure S2 — Probenecid-mediated inhibition of Panx1 activity in HeLa cells. Cells were mock treated (unstimulated) or water stimulated in the presence (stimulated+probenecid) or absence (stimulated) of 1.0 mM probenecid. Live-cell phase contrast and corresponding fluorescence images were taken 1 min after addition of YoPro-1 iodide. (TIF) [file pone.0063732.s002.tif]

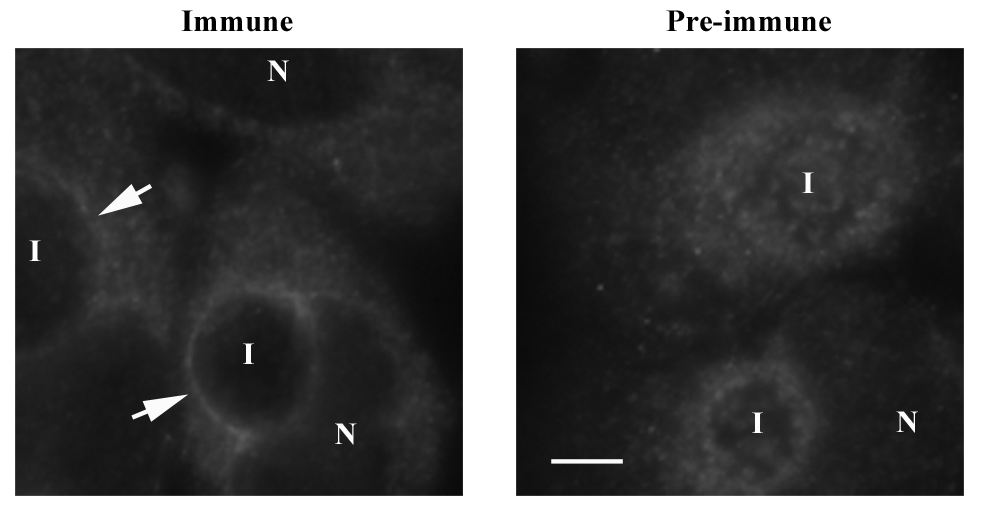

Supplement: Figure S3 — Immunolocalization of endogenous Panx1 in C. trachomatis infected cells. HeLa cells were infected at an MOI of 1 and fixed 24 hr post infection. Parallel samples were probed with chicken anti-pannexi1 (Immune) or matched pre-immune (Pre-immune) serum. Panx1 was visualized via epi-fluorescence microscopy after probing with Alexa594-coupled secondary antibodies. Images were acquired at 90X magnification and Bar = 5 µm. Chlamydial inclusions (I), host nuclei (N), or Panx1 colocalization with inclusions (arrows) are indicated. Pre-immune serum alone resulted in detection of intracellular chlamydiae. (TIF) [file pone.0063732.s003.tif]
